# The Mutant p53-Driven Secretome Has Oncogenic Functions in Pancreatic Ductal Adenocarcinoma Cells

**DOI:** 10.3390/biom10060884

**Published:** 2020-06-09

**Authors:** Giovanna Butera, Jessica Brandi, Chiara Cavallini, Aldo Scarpa, Rita T. Lawlor, Maria Teresa Scupoli, Emílio Marengo, Daniela Cecconi, Marcello Manfredi, Massimo Donadelli

**Affiliations:** 1 Department of Neurosciences, Biomedicine and Movement Sciences, Section of Biochemistry, University of Verona, Strada Le Grazie 8, 37134 Verona, Italy; giovanna.butera@univr.it (G.B.); mariateresa.scupoli@univr.it (M.T.S.); 2 Department of Biotechnology, University of Verona, 37134 Verona, Italy; jessica.brandi@univr.it (J.B.); daniela.cecconi@univr.it (D.C.); 3 Research Center LURM (Interdepartmental Laboratory of Medical Research), University of Verona, 37134 Verona, Italy; chiara.cavallini@univr.it; 4 Department of Diagnostics and Public health, Section of Pathology, University of Verona, 37134 Verona, Italy; aldo.scarpa@univr.it; 5 ARC-Net Centre for Applied Research on Cancer, University and Hospital Trust of Verona, 37134 Verona, Italy; rita.lawlor@arc-net.it; 6 Department of Sciences and Technological Innovation, University of Piemonte Orientale, 28100 Novara, Italy; emilio.marengo@uniupo.it; 7 Center for Translational Research on Autoimmune and Allergic Diseases, University of Piemonte Orientale, Italy, ISALIT, Spin-off at the University of Piemonte Orientale, 28100 Novara, Italy; 8 Department of Translational Medicine, University of Piemonte Orientale, Italy, CAAD, corso Trieste 15/A, 28100 Novara, Italy

**Keywords:** mutant p53, pancreatic adenocarcinoma, secretome, proteomics, oncogenes, gain-of-function

## Abstract

The cancer secretome is a rich repository of useful information for both cancer biology and clinical oncology. A better understanding of cancer secretome is particularly relevant for pancreatic ductal adenocarcinoma (PDAC), whose extremely high mortality rate is mainly due to early metastasis, resistance to conventional treatments, lack of recognizable symptoms, and assays for early detection. TP53 gene is a master transcriptional regulator controlling several key cellular pathways and it is mutated in ~75% of PDACs. We report the functional effect of the hot-spot p53 mutant isoforms R175H and R273H on cancer cell secretome, showing their influence on proliferation, chemoresistance, apoptosis, and autophagy, as well as cell migration and epithelial-mesenchymal transition. We compared the secretome of p53-null AsPC-1 PDAC cells after ectopic over-expression of R175H-mutp53 or R273H-mutp53 to identify the differentially secreted proteins by mutant p53. By using high-resolution SWATH-MS technology, we found a great number of differentially secreted proteins by the two p53 mutants, 15 of which are common to both mutants. Most of these secreted proteins are reported to promote cancer progression and epithelial-mesenchymal transition and might constitute a biomarker secreted signature that is driven by the hot-spot p53 mutants in PDAC.

## 1. Introduction

Pancreatic ductal adenocarcinoma (PDAC) is the most common type of pancreatic cancer; it is characterized by poor prognosis, with a dismal overall five-year survival rate of ~5% [1,2]. A typical feature of PDAC is the lack of early phase specific symptoms that leads to a late-stage diagnosis. In addition, the absence of early biomarkers, detection methods, and chemoresistance are the main drivers of poor prognosis in PDAC [3,4]. Chemoresistance is primarily caused by key genetic alterations, which favour disorders in the apoptotic pathway [3]. One of the most important proteins involved in DNA damage repair and apoptosis is the tumor suppressor protein p53 (TP53), which regulates a wide range of cellular biological processes to prevent tumor formation by killing or delaying the growth of neoplastic cells [5]. The importance of the p53 pathway in tumor suppression is also highlighted by the observation that *TP53* mutations are associated to poor prognosis [6] and they are present in about half of all human cancers, reaching even ~75% of PDAC patients [7,8]. The great majority of p53 alterations are missense mutations that are localized in the DNA binding domain, which result in the expression of full-length mutant p53 isoforms [9]. The most frequent p53 alterations are missense mutations in the DNA binding domain (DBD), called hot-spot mutants, which cause the expression of full-length p53 mutant isoforms. These mutations in the DBD are grouped into two main types: conformational mutations, such as mutp53-R175H, and contact mutations, such as mutp53-R273H, which cause structural modifications in the binding domain or affect the DNA binding ability of the protein, respectively [10]. Both kinds of mutations alter p53’s interaction with its consensus DNA-binding sequence, negatively impacting the activation of tumor suppressor wild type-p53 target genes.

In addition, these mutants can acquire new oncogenic functions and they are named gain-of-function (GOF) mutants. In fact, although they lose the capability to bind DNA and regulate wtp53-target genes, they can regulate the transcription of a different set of genes that induce cancer aggressiveness. This is achieved through direct interaction with various transcription factors or repressors in the transcriptional complex. This results in the development of the typical hallmarks of cancer cells carrying the mutant *TP53* gene, such as chemoresistance [11], metabolic alterations [12,13], and genomic instability [14]. Furthermore, mutant p53 isoforms strongly accumulate in cells as a result of a reduction in the rate of mutant p53 protein degradation due to its inability to induce the E3 ubiquitin-protein ligase MDM2 [15], thus amplifying the oncogenic effects of the protein.

Many recent studies reveal the role of p53 mutant proteins in the modification of the tumor microenvironment and secretome of cancer cells, altering the secretion of inflammatory cytokines, affecting the crosstalk between cancer and stromal cells, and increasing the extracellular acidification [16,17,18]. Cancer aggressiveness is strongly dependent on the composition of the extracellular microenvironment, which is itself affected by the release of proteins by the cancer cells. Indeed, secreted proteins may promote carcinogenesis, favoring key roles, such as cell signaling, communication and migration [19,20]. Thus, the secretome of cancer cells represents an unique opportunity to collect and identify several secreted macromolecules and may be considered a valuable source for biomarker discovery and the identification of novel therapeutic targets [18,21].

In the present study, we investigate the functional effect of mutp53-driven secretome of PDAC cells, demonstrating its impact on several hallmarks of cancer cells carrying the mutant *TP53* gene, such as hyper-proliferation, chemoresistance, inhibition of apoptosis and autophagy, cell migration, and epithelial-to-mesenchymal transition. In order to identify a mutp53-dependent signature of secreted proteins by PDAC cells, a proteomics approach has been used. We identified 15 hypo- or hyper-secreted proteins in common to both R175H and R273H hot-spot mutant p53 isoforms. These results definitively clarify the functional impact of mutp53-driven secretome in PDAC aggressiveness and provide crucial insights on the identification of mutp53-dependent PDAC secretome.

## 2. Materials and Methods

### 2.1. Chemicals

Gemcitabine (2′,2′-difluoro-2′-deoxycytidine; GEM) was provided by Accord Healthcare (Milan, Italy) and it was solubilized in sterile water.

### 2.2. Cell Culture

PDAC cell line AsPC-1 (p53-null) was grown in RPMI 1640, while lung cancer cell line H1299 (p53-null) was cultured in DMEM medium (Life Technologies, Milan, Italy). Both culture media were supplemented with 10% FBS, and 50 μg/mL gentamicin sulfate (BioWhittaker, Lonza, Bergamo, Italy). AsPC1 was purchased by ATCC (Manassas, VA, USA), while both of the mock clone and clone stably expressing mutant p53-R273H of the p53-null H1299 cells were kindly provided by Dr. Riccardo Spizzo (Centro di Riferimento Oncologico, National Cancer Institute, Aviano, Italy). The adherent cells were incubated at 37 °C with 5% CO_2_.

### 2.3. Transient Transfection Assay

AsPC-1 cells were seeded in 96-well or in six-well plates. Wild type or mutant p53 ectopic expression in p53-null cancer cells was obtained by transfecting pcDNA3-mutp53R273H, pcDNA3-mutp53R175H, or pCMV-wild type p53 expression vectors or their relative negative control (pcDNA3 or pCMV). Transfections were carried out using Lipofectamine 3000 (Thermo Fisher Scientific, Milan, Italy) for 48 h, according to the manufacturer’s instructions.

### 2.4. Cell Proliferation Assay

AsPC-1 cells were seeded in 96-well plates (8 × 10^3^ cells/well). Forty-eight hours later, cell growth was measured by Crystal Violet assay (Sigma, Milan, Italy) according to the manufacturer’s protocol, and the absorbance was measured by spectrophotometric analysis (A_595_ nm).

### 2.5. Apoptosis Assay

The cells were seeded in 96-well plates (8 × 10^3^ cells/well). Forty-eight hours later, cells were fixed with 2% paraformaldehyde in PBS for 15 min. at room temperature, washed twice with PBS, and then stained with annexinV/FITC (Bender MedSystem, Milan, Italy) in binding buffer (10 mM HEPES/NaOH pH 7.4, 140 mM NaCl, and 2.5 mM CaCl_2_) for 10 min. at room temperature in the dark. The cells were then washed with binding buffer and fluorescence was measured using a multimode plate reader (Ex_485_ nm and Em_535_ nm) (GENios Pro, Tecan, Milan, Italy). The values were normalized on cell proliferation by Crystal Violet assay.

### 2.6. Autophagosome Formation Assay

The cells were stained with the fluorescent probe monodansylcadaverine (MDC; Sigma, Milan, Italy) to quantify the induction of autophagy, since MDC is a maker for acidic vesicular organelles (AVOs), as autophagic vacuoles and autolysosomes. Briefly, cells were seeded in 96-well plates (5 × 10^3^ cells/well) and, 48 h later, cells were incubated in culture medium containing 50 μM MDC at 37 °C for 15 min. Cells were then washed with Hanks buffer (20 mM Hepes pH 7.2, 10 mM glucose, 118 mM NaCl, 4.6 mM KCl, and 1 mM CaCl_2_) and fluorescence was measured using a multimode plate reader (Ex_340_ nm and Em_535_ nm) (GENios Pro, Tecan, Milan, Italy). The values were normalised on cell proliferation by Crystal Violet assay.

### 2.7. Wound-Closure Cell Migration Assay

AsPC-1 cells were seeded in six-well plate (6 × 10^5^ cells/well). A scratch was made across the center of the AsPC1 p53-null monolayer cells using a sterile 200-µL pipette tip. Subsequently, the cells were washed with PBS to remove the detached cells and incubated with conditioned medium (CM) released by transfected AsPC-1 cells for 48 h. Cell migration was observed in time-lapse (EVOS). Images of cells movement were captured every 2 h for 48 h and were further analyzed quantitatively using NIH ImageJ computing software (http://rsb.info.nih.gov/nih-image/). Migration ability as relative migration distance (RMD) was evaluated using the following formula: RMD (%) = 100 (A − B)/A, with A and B representing the width of cell scratches at time 0 and after 48 h of incubation, respectively.

### 2.8. FACS Analysis

The cells were trypsinized, washed with PBS, and then incubated with anti-human CD325 (N-Cadherin) antibody that was conjugated with PE (BioLegend, San Diego, CA, USA; Clone 8C11) or anti-human CD324 (E-Cadherin) antibody conjugated with PE (BioLegend; Clone 67A4). Unstained cells were used as negative control.

Approximately 10,000 gated events were acquired for each sample on a FACSCanto cytometer (Becton Dickinson, Franklin Lakes, NJ, USA). Flow cytometry data were analyzed using FlowJo software (v10; TreeStar, Ashland, OR, USA). Dead cells and debris were excluded on the basis of forward-scatter and side-scatter. N- and E-Cadherin expression was calculated as fold change: median fluorescence intensity (MFI) of stained sample normalized with respect to the MFI of unstained sample.

### 2.9. Immunoblot Analysis

The cells were harvested, washed in PBS, and solubilized in lysis buffer in the presence of phosphatase and protease inhibitors (50 mM Tris–HCl pH 8, 150 mM NaCl, 1% Igepal CA-630, 0.5% Na-Doc, 0.1% SDS, 1 mM Na_3_VO_4_, 1 mM NaF, 2.5 mM EDTA, 1 mM PMSF, and 1× protease inhibitor cocktail). After incubation on ice for 30 min., the lysates were centrifuged at 14,000× *g* for 10 min. at 4 °C and the supernatant fractions were used for Western blot analysis. Protein concentration was measured by Bradford reagent (Pierce, Milan, Italy) using bovine serum albumin as a standard. The protein extracts (20 μg/lane) were resolved on a 12% SDS-polyacrylamide gel and electro-blotted onto PVDF membranes (Millipore, Milan, Italy). After transferring proteins onto PVDF membranes, Amido Black 1X Staining Solution (Sigma–Aldrich #A8181) was used to confirm equal protein loading in different lanes. Briefly, the membranes were covered with Amido Black and stain by gentle shaking for one minute at room temperature; after that, membranes were de-stained by placing in an aqueous solution containing 25% isopropanol and 10% acetic acid for 30 minutes at room temperature. The membranes were blocked in 5% low-fat milk in TBST (50 mM Tris pH 7.5, 0.9% NaCl, 0.1% Tween 20) for 1 h at room temperature and probed overnight at 4 °C with a mouse polyclonal anti-p53 (1:2000) (Santa Cruz, #sc-263), rabbit monoclonal anti-glyceraldehyde 3-phosphate dehydrogenase (GAPDH) (1:1000) (Cell Signaling, #5174S) antibodies. Mouse monoclonal anti-vimentin (1:200) (Santa Cruz, #sc-373717) was used to detect the expression levels of secreted vimentin in protein extracts that were derived from conditioned medium by a protein extraction method described in the next paragraph. Horseradish peroxidase conjugated anti-mouse or anti-rabbit IgGs (1:8000 in blocking solution) (Upstate Biotechnology, Milan, Italy) were used as secondary antibodies. Immunodetection was carried out using chemiluminescent substrates (Amersham Pharmacia Biotech, Milan, Italy) and recorded using a HyperfilmECL (Amersham Pharmacia Biotech). The ECL (Enhanced ChemiLuminescence) results were scanned and the amount of each protein band was quantified using NIH Image J software.

### 2.10. Protein Extraction from Conditioned Medium (CM)

The day after transient transfection, the AsPC-1 cells were washed six times in PBS (phosphate-buffered saline) and then incubated in serum-free RPMI for 22 h. This serum-free time period of incubation has been chosen on the basis of our previous investigations in order to avoid cell injury. Cell viability, as determined with 0.4% trypan blue solution (Thermo Fischer Scientific), was higher than 95%. The media containing secreted proteins were collected by centrifugation at 1,000× *g* for 10 min. to pellet floating cells and were defined as conditioned media (CM). After the addition of 1× protease inhibitor cocktail (Roche), CM were centrifuged again at 17,000× *g* for 20 min. at 4 °C to pellet the remaining cell debris. The proteins in the CM were precipitated overnight at −20 °C with 4 volumes of ice-cold acetone. The pellets were then collected by centrifugation at 17,000× *g* for 20 min. at 4 °C and then resuspended in 100 mM ammonium bicarbonate (NH_4_HCO_3_). Protein concentrations were determined using BCA protein assay (Sigma).

### 2.11. In-Solution Digestion

Before SWATH-MS analysis, the CM proteins were digested following the protocol provided by the manufacture (Applied Biosystem). Briefly, samples were prepared to have 100 µg of protein in a final volume of 25 µL of 100 mM NH_4_HCO_3_. The proteins were reduced using 2.5 µL of dithiothreitol (200 mM DTT stock solution) (Sigma) at 90 °C for 20 min. and alkylated with 10 µL of Cysteine Blocking Reagent (Iodoacetamide, IAM, 200 mM; Sigma) for 1 h at room temperature in the dark. DTT stock solution was then added to destroy the excess of IAM. After dilution with 300 µL of water and 100 µL of NH_4_HCO_3_ to raise pH, 5 µg of trypsin (Promega, Sequence Grade) was added and digestion was performed overnight at 37 °C. Trypsin activity was stopped by adding 2 µL of neat formic acid and the digests were dried by Speed Vacuum [21].

### 2.12. Data Acquisition

The digested samples were analyzed on a micro-LC Eksigent Technologies (Dublin, OH, USA) interfaced to a 5600+ TripleTOF mass spectrometer system (AB Sciex, Concord, ON, Canada) that was equipped with a DuoSpray Ion Source and a CDS (Calibrant Delivery System). The LC column was a Halo Fused C18 (AB Sciex, Concord, ON, Canada). The mobile phase was a mixture of 0.1% (*v*/*v*) formic acid in water (A) and 0.1% (*v*/*v*) formic acid in acetonitrile (B), eluting at a flow-rate of 15.0 μL min.^−1^ at an increasing concentration of solvent B from 2% to 40% in 30 min. The injection volume was 4.0 μL and the oven temperature was set at 40 °C. For identification purposes, the samples were subjected to data dependent analysis (DDA): the mass spectrometer operated using a mass range of 100–1500 Da (TOF scan with an accumulation time of 0.25 s), followed by a MS/MS product ion scan from 200 to 1250 Da (accumulation time of 5.0 ms) with the abundance threshold set at 30 cps (35 candidate ions can be monitored during every cycle). The ion source parameters in electrospray positive mode were set, as follows: curtain gas (N2) at 25 psig, nebulizer gas GAS1 at 25 psig, and GAS2 at 20 psig, ionspray floating voltage (ISFV) at 5000 V, source temperature at 450 °C, and declustering potential at 25 V. For the quantification, the samples were subjected to cyclic data independent analysis (DIA) of the mass spectra, using a 25-Da window: the mass spectrometer was operated, such that a 50-ms survey scan (TOF-MS) was performed and subsequent MS/MS experiments were performed on all precursors. These MS/MS experiments were performed in a cyclic manner while using an accumulation time of 40 ms per 25-Da swath (36 swaths in total) for a total cycle time of 1.5408 s [22]. The ions were fragmented for each MS/MS experiment in the collision cell while using the rolling collision energy. The MS data were acquired with Analyst TF 1.7 (AB SCIEX, Concord, ON, Canada). Two DDA and three DIA acquisitions were performed.

### 2.13. Protein Database Search

The DDA files were searched using Protein Pilot software v. 4.2 (AB SCIEX, Concord, ON, Canada) and Mascot v. 2.4 (Matrix Science Inc., Boston, MA, USA). The DIA files were converted to pseudo-MS/MS spectra with DIA-Umpire software and they were searched as DDA files [23,24]. Trypsin as digestion enzyme was specified for both the software. For Mascot we used two missed cleavages, the instrument was set to ESI-QUAD-TOF, and the following modifications were specified for the search: carbamidomethyl cysteins as fixed modification and oxidized methionine as variable modification. A search tolerance of 0.08 Da was specified for the peptide mass tolerance, and 10 ppm for the MS/MS tolerance. The charges of the peptides to search for were set to 2 +, 3 +, and 4 +, and the search was set on monoisotopic mass. The UniProt Swiss-Prot reviewed database containing human proteins (version 2015.07.07, containing 42131 sequence entries) was used and a target-decoy database search was performed. False Discovery Rate was fixed at 1%.

### 2.14. Protein Quantification

MS1 (precursor ion masses) and MS2 (fragment ion masses) chromatogram based quantitation was carried out in Skyline 3.1, an open source software project (http://proteome.gs.washington.edu/software/skyline) [25]. Spectral libraries were generated in Skyline from database searches. All of the raw files acquired in DIA were directly imported into Skyline and MS1 precursor ions and MS/MS fragment ions were extracted for all peptides present in the MS/MS spectral libraries. Quantitative analysis was based on extracted ion chromatograms (XICs) for the top three resulting precursor ion peak areas e.g., M, M + 1, and M + 2 (MS1) and on XICs of up to three MS/MS fragment ions, typically y- and b-ions, matchingspecific peptides present in the spectral libraries. For statistical analysis of quantitative differences of proteins and peptides between samples, MSstats (v.2.0), an open-source R-based package [26], was used.

### 2.15. Bioinformatics and Statistics Software

The potential secretion pathways of regulated proteins were predicted with the SecretomeP 2.0 server (http://www.cbs.dtu.dk/services/SecretomeP/) for classical and non-classical secretion, while the localization of signal peptide cleavage sites were predicted with SignalP v.5.0 (http://www.cbs.dtu.dk/services/SignalP/) [27].

The regulated proteins were analyzed using the STRING database (v.11.0) (http://string-db.org), which is a database of known and predicted protein-protein interactions [28]. The Cytoscape (v.3.7.2) ClueGO (v.2.5.4) plugin was used to visualize the enriched pathways associated with the Kyoto Encyclopaedia of Genes and Genome (KEGG) database [29]. In brief, KEGG pathways were explored with medium specificity and a kappa score of 0.4. An enrichment/depletion method with a two-sided hypergeometric test was applied, correct with the Bonferroni step down for each p-value calculation. Enriched pathways with a *p*-value < 0.05 were considered to be significant. Functional annotation of identified proteins was employed while using the Database for Annotation, Visualization, and Integrated Discovery (DAVID) (v.6.8) (http://david.abcc.ncifcrf.gov/) to identify gene ontology (GO) biological processes (BPs), molecular function (MFs), and cellular component (CCs). The BPs, MFs, and CCs that were enriched by the list of proteins were identified as the ones with *p*-value < 0.01 calculated by DAVID [30].

### 2.16. Statistical Analysis

ANOVA analysis was performed by GraphPad Prism 5 software. *p* value < 0.05 was indicated as being statistically significant. Values are the means of three independent experiments (± SD).

## 3. Results

### 3.1. Cancer Cell Secretome Driven by mutant p53 Induces Hyper-Proliferative Effects

**Figure 1:**
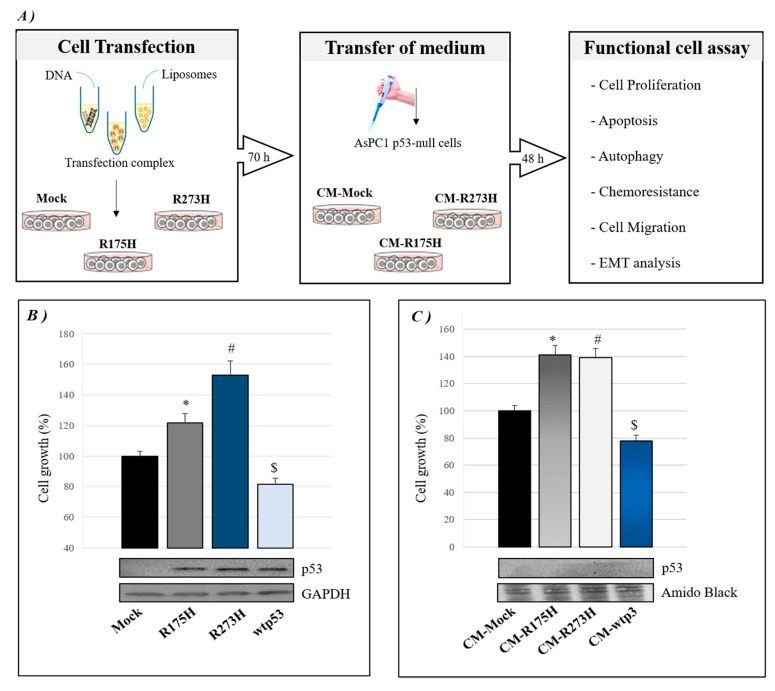
Cancer cell secretome driven by mutant p53 can induce hyper-proliferative effects. (**A**) A summary model of the approach used in this study: p53-null AsPC-1 cells were transfected with plasmids for R273H or R175H mutant p53 over-expression or its mock vector for 48 h. Then, AsPC-1 transfected cells were washed in PBS to remove liposomes and incubated with fresh culture medium for further 22 h to accumulate secreted proteins. After that, the conditioned medium (CM) of AsPC-1 transfected cells was transferred to untransfected p53-null AsPC-1 cells. After 48 h, several biological phenomena listed in the figure were investigated in AsPC-1 cells bearing mutp53-driven CM. (**B**) Cell growth was measured by Cristal Violet assay in p53-null AsPC-1 cells transfected for over-expression of wtp53, R175H or R273H mutp53. Accompanying Western blotting of p53 and of GAPDH for control loading are reported. Statistical analysis * *p* < 0.05 R175H vs Mock; # *p* < 0.05 R273H vs Mock; $ *p* < 0.05 wtp53 vs Mock. (**C**) Cell growth was measured by Cristal Violet assay in untransfected p53-null AsPC-1 cells cultivated with wtp53-, R175H- or R273H-CM. Accompanying Western blotting of p53 and amido black staining are reported. Statistical analysis * *p* < 0.05 CM-R175H vs CM-Mock; # *p* < 0.05 CM-R273H vs CM-Mock; $ *p* < 0.05 wtp53 vs CM-Mock.

We aimed to study the role of mutp53-driven secretome in cancer cell aggressiveness to investigate whether mutant p53 may influence the secretome of PDAC cells. We induced the exogenous expression of R273H or R175H mutp53 isoforms in p53-null AsPC-1 PDAC cells by using liposome-mediated transient transfection assay, as summarized in Figure 1A. Forty-eight hours later, we checked the effective over-expression of p53 in AsPC-1 by Western blotting and functionally analyzed the hyper-proliferative effect induced by mutant p53 isoforms, as compared to mock or wt-p53 (Figure 1B). Subsequently, AsPC-1 transfected cells were washed in PBS and then incubated in fresh culture medium for further 22 h to accumulate secreted proteins. This conditioned medium (CM) released by transfected AsPC-1 cells was transferred to new p53-null AsPC-1 cells, which were thus cultivated in wtp53- or mutp53-driven secretome for 48 h in order to study the functional effects of secretome driven by GOF R175H and R273H mutp53 isoforms. Figure 1C shows that both R175H and R273H p53 mutants are able to induce AsPC-1 cell hyper-proliferation through their mutp53-driven CM, as compared to their negative mock control or to wtp53-CM. Interestingly, in accordance with the tumor suppressor role of wild type p53, the wtp53-driven CM showed an inhibitory effect on cell growth. The absence of extracellular p53 in mutp53- or wtp53-driven CM was proved by Western blotting and then further confirmed by mass spectrometry analysis. Altogether, these data demonstrate that GOF mutant p53 isoforms can also exert their hyper-proliferative effects on cancer cells through the alteration of their secretome.

### 3.2. Mutp53-Driven Secretome Mediates Anti-Apoptotic, Anti-Autophagic and Chemoresistance Effects

**Figure 2:**
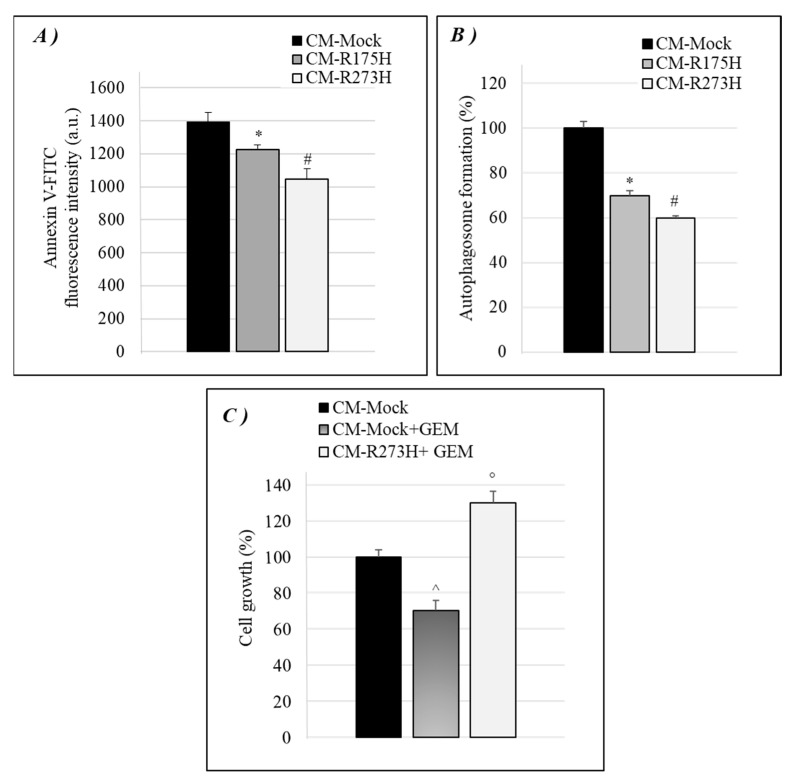
Mutp53-driven secretome mediates anti-apoptotic, anti-autophagic and chemoresistance effects. (**A**) Apoptosis was determined by the annexinV/FITC binding assay in AsPC-1 cultivated with mutp53-derived CM. The annexinV-FITC fluorescence intensity was measured by using a multimode plate reader and reported as arbitrary units (a.u.). (**B**) Autophagosome formation assay was determined by intracellular staining using the MDC probe in AsPC-1 cultivated with mutp53-derived CM. Statistical analysis * *p* < 0.05 CM-R175H vs CM-Mock; # *p* < 0.05 CM-R273H vs CM-Mock. (**C**) Cell growth was analyzed by Cristal Violet assay in AsPC-1 cultivated with mutp53-derived CM treated with 1 μM GEM for 48 h. Statistical analysis ^ *p* < 0.05 CM-R273H + GEM vs CM-Mock + GEM; ° *p* < 0.05 CM-Mock + GEM vs CM-Mock.

We tested whether CM-R175H and CM-R273H were also able to induce other typical hallmarks induced by intracellular GOF mutant p53 isoforms, as the inhibition of cell death-related phenomena (i.e., apoptosis and autophagy) and the stimulation of chemoresistance to the standard drug gemcitabine to better investigate the effects of mutant p53 secretome. Figure 2A shows that mutp53-derived CM was able to counteract apoptosis in p53-null AsPC-1 cells, in accordance with the well-known antiapoptotic effect of intracellular mutant p53 isoforms [31], which indicated that mutant p53 is also able to counteract apoptosis by promoting the secretion of some molecules that discourages cell death.

Moreover, since we previously demonstrated that GOF mutant p53 proteins blocked the autophagic flux by the regulation of some molecular pathways, as AMPK, AKT/mTOR, and some crucial autophagy-related genes (ATGs) [32,33], we wondered whether mutant p53 might also counteract autophagy by secretome alteration. Figure 2B shows that the CM released by R175H- or R273H-mutp53 AsPC-1 cancer cells decreases the amount of intracellular autophagic vesicles, when compared to p53-null driven CM.

Furthermore, our previous data showed that mutant p53 can stimulate the chemoresistance of PDAC cells to the drug gemcitabine (GEM) [11]. Thus, we investigated whether mutp53-driven secretome may also be able to reduce cancer cells sensitivity to GEM. Figure 2C shows that GEM inhibited cell growth of AsPC-1 cells that were cultivated with mock-derived CM, while the CM derived by R273H mutant p53 AsPC-1 cells counteracted the therapeutic effect of GEM, as compared to its mock control, representing an important aspect to be further considered for clinical therapeutic studies. Overall, these data provide evidence that mutant p53 proteins influence the secretion of components that contribute to cancer cell growth and resist cell death-related phenomena, such as apoptosis, autophagy, and anticancer drug exposure.

### 3.3. Mutp53-Driven Secretome Stimulates Cancer Cell Migration and Epithelial-to-Mesenchymal Transition

**Figure 3:**
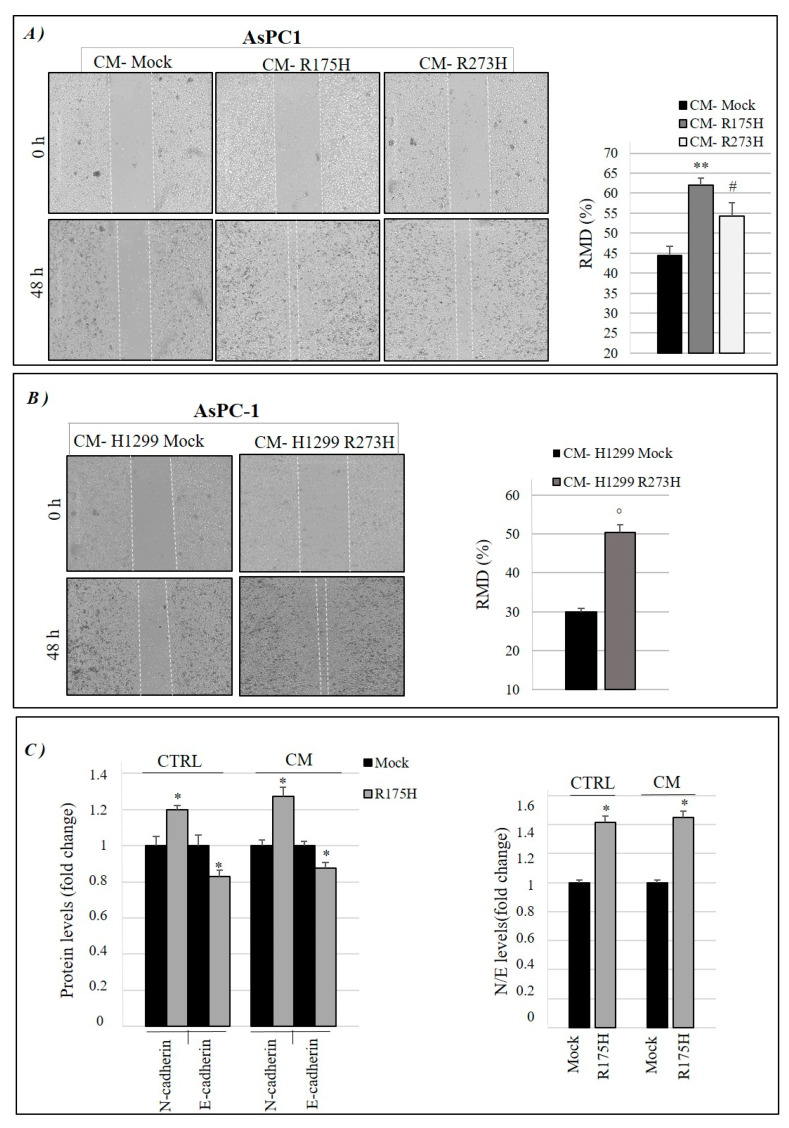
Mutp53-driven secretome stimulates cancer cell migration and epithelial-mesenchymal transition (EMT). (**A**) Wound closure cell assay on the confluent p53-null AsPC-1 cell monolayer that received mutp53-driven secretome from transiently transfected AsPC-1 cells or (**B**) from H1299 cells stably expressing mutant p53-R273H, as compared to its mock control. A scratch was performed in the cell monolayer at time zero, after that we monitored cell migration for 48 h. The images were analyzed quantitatively by using ImageJ computing software. Migration ability expressed as relative migration distance (RMD) increased in cells cultured with mutp53-derived CM. Statistical analysis ** *p*  <  0.01 CM-R175H vs CM-Mock; # *p*  <  0.05 CM-R273H vs CM-Mock; ° *p* < 0.05 CM-H1299 R273H vs Mock. (**C**) Quantification of N-cadherin and E-cadherin expression levels by FACS analysis in AsPC-1 cells transfected with mock or with plasmid for mutp53 R175H over-expression (CTRL) and in AsPC-1 cells bearing the conditioned medium from mock or mutp53 R175H-expressing AsPC-1 cells (CM) (left panel). We also calculated the expression level of N- to E-cadherin ratio (right panel). Statistical analysis * *p*  <  0.05 R175H vs mock.

Because GOF mutant p53 isoforms can stimulate cancer cell migration, we also investigated whether mutp53-induced modulation of secretome can have a role in this phenomenon [17]. Using the same methodological approach described in Figure 1A, we discovered that CM-R175H and CM-R273H are able to favor the migration of AsPC-1 cells (Figure 3A). In particular, we observed a faster wound closure in p53-null ASPC-1 cells cultivated with R175H or R273H mutant p53-driven secretome, as compared to CM derived from their mock control (CM-mock). We also used stable clones of p53-null lung cancer H1299 cells non-expressing (mock) or constitutively expressing the R273H mutant p53 isoform, which were previously used as a valuable cell model to study the oncogenic effects of mutant p53, in order to further straighten these data [33]. As control of mutant p53 expression and functionality, in Figure S1 we report the cellular hyper-proliferative effect and the mutant p53 expression level observed in mock and R273H-mutp53 clones of H1299 cells. Concerning the migration assay, Figure 3B shows also that the secretome derived by H1299 cancer cells stably expressing R273H mutant p53 (CM-H1299 R273H) induced cell migration of AsPC-1 p53-null cells as compared to mock control (CM-H1299 mock), further supporting the results on AsPC-1 secretome shown in Figure 3A.

Finally, we investigated whether GOF mutp53-driven secretome contributes to another typical feature that is induced by mutant p53, namely epithelial-to-mesenchymal transition (EMT) of cancer cells, which is a phenomenon strictly connected to cancer cell migration [34,35]. In Figure 3C, we show that R175H mutant p53 increased N-cadherin and decreased E-cadherin protein expression in cancer cell membrane, as compared to mock, thus markedly altering the N/E cadherin ratio, a typical feature of EMT. Intriguingly, CM-derived by AsPC-1 overexpressing R175H mutant p53 is also able to induce the same regulation of N- and E-cadherin expression level in cancer cell membrane, as compared to CM-mock derived by AsPC-1 cells. Consequently, the N/E cadherin ratio was higher in AsPC-1 cells cultivated with CM-R175H as compared to the same cells that were cultivated with CM-mock. Since N-cadherin promotes motility, invasion, and produces a scattered phenotype with EMT, in association with a reduction in the expression of E-cadherin [36,37], our results suggest that proteins that are secreted by mutant p53-expressing cancer cells have a crucial role in EMT transition, thus sustaining the migration and aggressiveness of cancer cells.

### 3.4. Identification of Secreted Proteins from Mutp53-Driven Secretome

After the investigation of the oncogenic functions of mutp53-driven secretome, we aimed to identify the main differentially secreted proteins by mutant p53 isoforms in PDAC. Thus, we analyzed the protein composition of the CM released by AsPC-1 cells expressing GOF mutp53 as compared to p53-null AsPC-1 cells (mock). After the transfection period (48 h), the cells were washed to remove DNA:liposome complexes and they were cultured for a further 22 hours to accumulate secreted proteins in serum-free culture medium to avoid protein cross-contamination by serum. Notably, this serum-free culture period has been identified as the maximum time period without delay of cell growth or signals of cell death (Figure S2) to avoid undiscriminating cellular lysis. A peptide liquid chromatography separation followed by mass spectrometry analysis and database search with Protein Pilot and Mascot was then performed. SWATH-MS analyses were performed in triplicates for each analyzed sample and they were imported in the Skyline software to perform the label-free quantification and the identification of regulated proteins. We identified 194 proteins in CM-R273H and 228 proteins in CM-R175H transfected cells. The major part of them (165 proteins) were in common between the two mutp53-driven secretomes of AsPC-1 cells. The semi-quantitative proteomic analysis showed that 45 proteins resulted in being significantly modulated in the CM-R175H (Table S1) and 58 proteins were significantly modulated in CM-R273H (Table S2) as compared to the mock control (CM-mock). Further analyzing the modulated proteins, we identified 15 differentially secreted proteins in common between CM-R175H and CM-R273H (Table 1**)**. Among these proteins that can better represent a common signature of secretome alteration driven by different GOF p53 mutant isoforms, there are several interesting proteins, such as growth factors (IGFBP1), histone proteins (HIST1H3A, HIST1H4A, HIST2H2AA3, HIST2H3A, and H2AFV), endothelial protein C receptor (EPCR), and others, which are commented in the Discussion section. We analyzed by western blotting the extracellular expression level of vimentin (VIM) in CM-mock, CM-R273H, and CM-R175H of AsPC-1 cells, confirming the hyper-secretion of VIM induced by both mutant p53 isoforms in order to validate mass spectrometry data (Figure S3). We have further investigated whether these 15 proteins were already identified in PDAC-derived exosomes and seven of them, namely PROCR, TIMP1, EZR, PSAP, VIM, CLSTN1 and CFL1, were detected in the proteome of pancreatic cancer exosomes using mass spectrometry [38], which suggests their roles as key signaling molecules.

### 3.5. Bioinformatic Analyses and Interaction Networks of Mutp53-Driven Secreted Proteins

Proteins that were detected in secretome samples were analyzed with the SignalP 5.0 and SecretomeP 2.0 prediction algorithms in order to determine which proteins are predicted to be secreted via classical (signal peptide-directed) or non-classical secretion mechanisms. The SignalP software allowed for defining the species that are secreted through the classical endoplasmic reticulum (ER)/Golgi pathway: all of the proteins lacking the presence of the classical signal peptide for the translocation to the ER, were then tested by SecretomeP 2.0 software for the putative export through one of the so-called non-classical secretory pathways (Table S3). Out of the 194 proteins that were detected in CM-R273H, secretory signal peptides were present in 42 (22%) of these proteins (SignalP D score >0.45) and 33 (17%) were identified as non-classically secreted proteins (SecretomeP NN score >0.6, with no secretory signal present). Of the 228 proteins that were detected in CM-R175H transfected cells, secretory signal peptides were present in 36 (16%) (SignalP D score >0.45) and 33 (15%) were identified as non-classically secreted proteins (SecretomeP NN score >0.6, with no secretory signal present). Together, these predictions account for ∼55% and ∼31% of the secreted proteins detected in CM of R273H and of R175H transfected AsPC-1 cells, respectively. Thus, these algorithms are not able to predict which subsets of proteins may be released from cells: in fact, proteins from various subcellular locations may be released by different mechanisms to play a biological role outside of the cell. This is also confirmed by the fact that, out of the 257 secreted proteins, 204 (79%) have been previously identified in cancer secretomes (www.cancersecretome.org).

**Figure 4:**
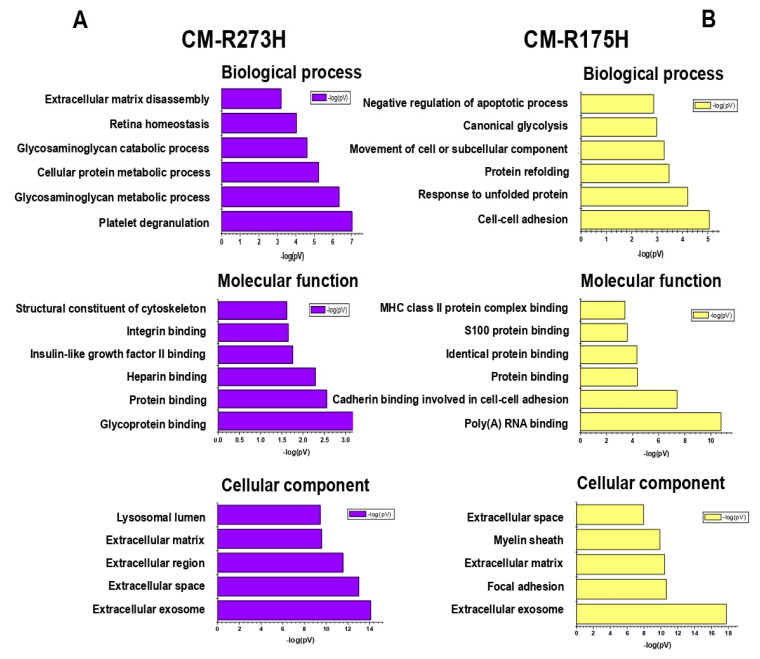
Bioinformatic analyses of enriched secreted proteins. Biological processes, molecular functions and cellular component of secreted proteins detected in (**A**) CM of R273H and in (**B**) CM of R175H transfected AsPC-1 cells.

The functional annotation of enriched secreted proteins was examined using DAVID software by performing enrichment analysis of biological processes (BPs), molecular functions (MFs), and cellular components (CCs) (Figure 4A,B).

Interestingly, the most significantly enriched BPs categories included cell-cell adhesion (22% of proteins), canonical glycolysis (9%), glycosaminoglycan metabolic process (13%) and extracellular matrix disassembly (10%). Moreover, the DAVID software associated the secretome profile with some interesting molecular functions, such as glycoprotein binding (10%) and insulin-like grow factor II binding (5%). Finally, the enrichment analysis of CCs revealed that enriched secreted proteins were mainly localized into the extracellular exosome, i.e., vesicles released in the extracellular region (87% and 74%), as well as in the extracellular matrix (Table S4).

**Figure 5:**
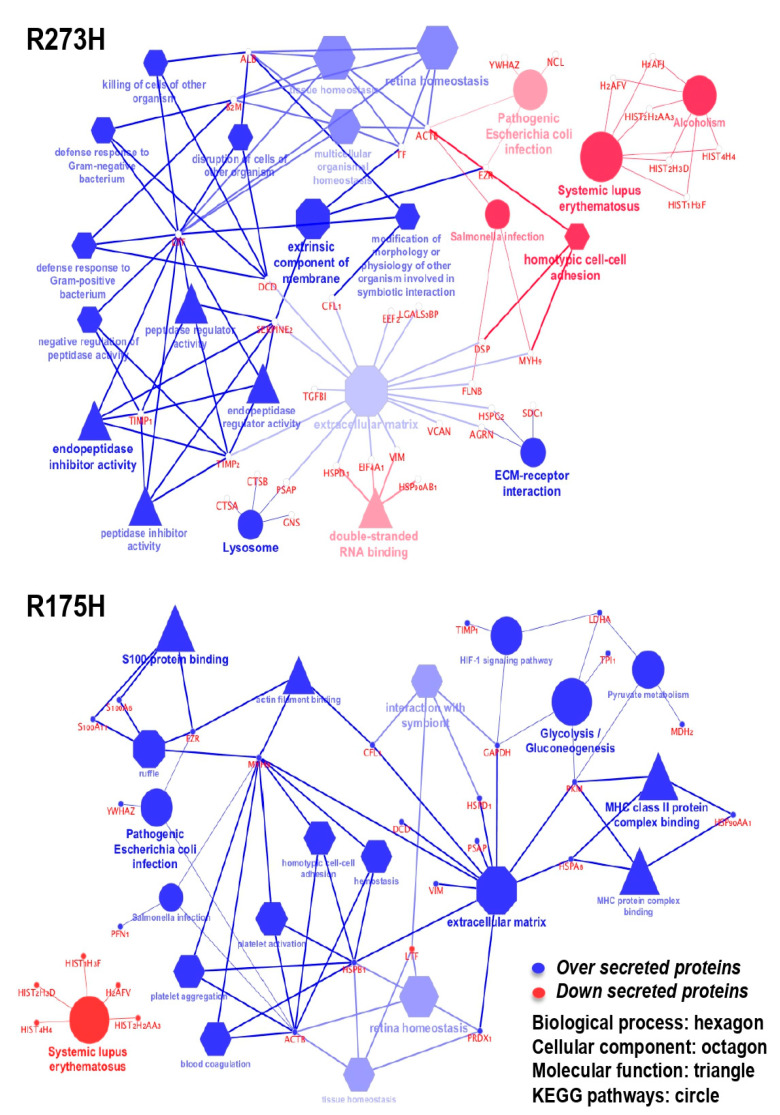
Cytoscape-based ClueGo/CluePedia pathway analysis and visualization. The enriched pathways were derived by the Kyoto Encyclopaedia of Genes and Genome (KEGG) database. The figure reports the functionally grouped networks of regulated proteins in the R273H and R175H transfected cell line. Blue term indicates an abundance increase compared to the mock control while red term indicates an abundance decrease. Terms are linked based on к-score (≥0.4), edge thickness indicates the association strength while node size corresponds to the statistical significance for each term. Biological processes are represented with hexagons, cellular components with octagons, molecular functions with triangles and KEEG pathways with circles.

We have further explored the enriched pathways and functions that are associated with modulated proteins quantified in the secretome by using the ClueGO app for Cytoscape platform. Only significant pathways or terms were presented by setting the statistical threshold (*p*-value < 0.05) and using the KEGG database as a reference. Figure 5 shows the functionally grouped networks of regulated proteins in the CM-R273H and CM-R175H samples. A blue term indicates an abundance increase when compared to the mock control, while a red term indicates an abundance decrease. Terms are linked based on к-score (≥0.4), edge thickness indicates the association strength while node size corresponds to the statistical significance for each term. The results show an over secretion of the endopeptidase inhibitor activity and the down-regulation of systemic lupus erythematosus related-proteins for CM-R273H. The analysis of CM-R175H sample reported an enrichment of proteins related to extracellular matrix (also present in CM-R273H) and an interesting enrichment of proteins that are involved in the glycolysis and gluconeogenesis, in agreement with the DAVID results.

**Figure 6:**
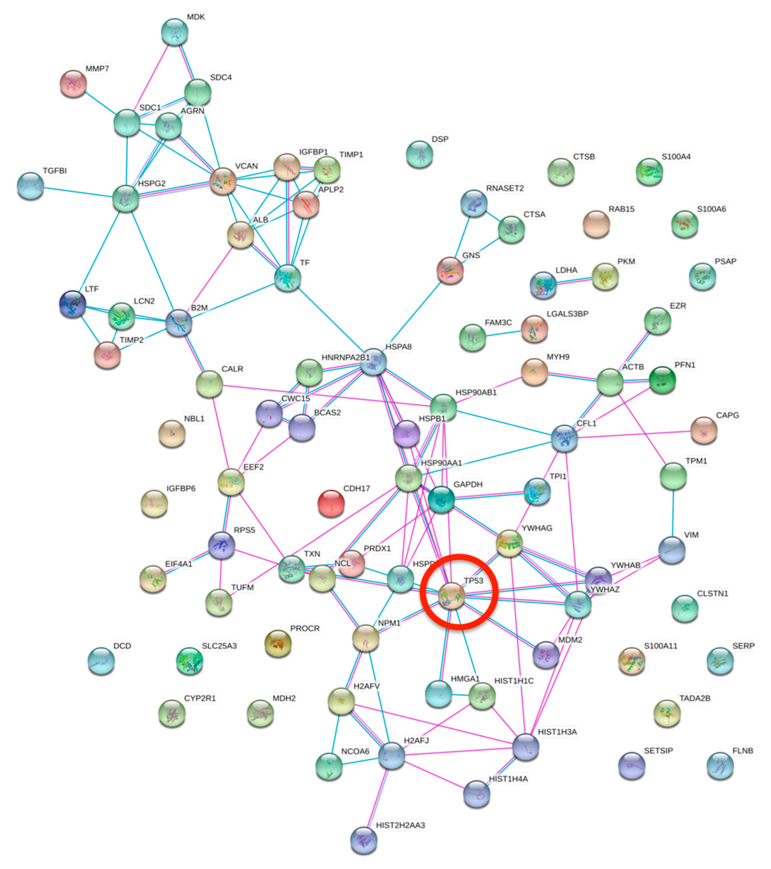
STRING analysis. Protein-protein interactions among modulated proteins secreted by both R273H and R175H transfected cells. TP53 was manually added to identify potentially related connections. Among the modulated proteins, 11 proteins are clustered in a tight interaction network centered on p53.

STRING software was employed in order to investigate the functional and physical protein interactions among p53 and the differentially secreted proteins (Figure 6). Our analysis revealed that the following 11 proteins are clustered in a tight interaction network centered on p53: HMGA1, HIST1H1C, YWHAZ, YWHAG, HSP90AB1, HSPB1, HSP90AA1, HSPA8, PRDX1, TXN, and NPM1. Altogether these data allow for better understanding the complex network of proteins differentially secreted by mutant p53 and their relative functions and potential interactions.

## 4. Discussion

The *TP53* gene is one of the most frequently mutated genes in cancers, especially in PDAC, and most of its mutations are missense mutations in the DNA binding domain, resulting in the expression of mutant isoforms of p53, which acquire new oncogenic properties, referred to as gain-of-function (GOF) [9]. These novel functions are involved in a plethora of different cellular pathways that are focused on cancer progression and aggressiveness, counteracting apoptosis, autophagy, and cellular senescence, and promoting invasion, metastasis, and chemoresistance [11,39,40]. The influence of mutant p53 in the clinical outcome of cancer patients, the high frequency of GOF mutations in the *TP53* gene, and the involvement of mutant p53 in a number of different cellular pathways have stressed the need to deeply investigate the events that are associated to cancer progression driven by mutant p53 isoforms in molecular oncology. Here, we discovered that the secretome driven by mutant p53 is able to favor cell growth and chemoresistance and counteract cell death-related mechanisms. Thus, we investigated the secreted proteins modulated by mutant p53 and identified a number of differentially secreted proteins by R175H (Supplementary Materials Table S1) or R273H (Supplementary Materials Table S2) mutant p53 isoforms. Interestingly, we identified 15 proteins (listed in Table 1) that were differentially secreted by both mutant p53 isoforms with the same trend of regulation. The clinical impact in cancers of these proteins and their involvement in the modulation of cancer microenvironment are discussed below.

Among mutp53-driven hyper-secreted proteins, we identified cofilin (COF1), which is involved in tumor cell migration and invasion by promoting actin cytoskeleton reorganization and cell-cell adhesion regulation and the level of cofilin-1 in patient’s sera is associated with PDAC progression and poor prognosis [41,42,43]; calsyntenin-1 (CLSTN1), which plays a fundamental role in cellular adhesion and cell communication and its expression level was increased in sera of patients with lung adenocarcinoma or in ovarian adenocarcinoma cell lines, but the molecular mechanisms of CLSTN1 in cancer still need to be investigated [44,45,46]; vimentin (VIME), which is a well-known marker for epithelial-mesenchymal transition (EMT), and elevated levels of circulating vimentin were detected in hepatocellular carcinoma and in circulating tumor cells (CTCs) in PDAC. Notably, the presence of vimentin^+^ CTCs was negatively associated with progression-free survival [47,48,49]. We also found that both R273H and R175H p53 mutant isoforms favor the hyper-secretion of the tissue inhibitor matrix metalloproteinase 1 (TIMP1) in PDAC cells. Matrix metalloproteases (MMPs) are able to impact tumor cell behavior in vivo by several means: (i) the direct degradation of the stromal connective tissue and basement membrane components, favoring the invasion and metastasis of cancer cells [50]; (ii) cleavage of membrane-bound growth factors or cytokines as well as their receptors [51]; (iii) and, cleavage of cell adhesion molecules, such as cadherins, leading to an increased cell motility occurring in EMT [52]. On the other hand, the activity of MMPs is specifically inhibited by TIMPs, but it is now assumed that TIMPs are multifactorial proteins that are also involved in the induction of proliferation and inhibition of apoptosis [53]. Eirò et al. found that subgroups of tumors showing a stromal molecular profile of abundant MMPs and TIMPs expression are strongly associated with higher recurrence of distant metastases. On the contrary, tumors with stromal phenotypes displaying low molecular profiles have an excellent clinical outcome [54]. These are relevant findings when considering that MMPs are mainly governed by the tumor stroma and they exert powerful influences on the local tissue microenvironment during tumorigenesis and progression. In this context, we suggest that TIMP1 hypersecretion by tumor cells expressing mutant p53 might further contribute to render the tumor microenvironment prone to invasion or metastasis. Accordingly, TIMP1 is considered as a prognostic marker for cancer progression and metastasis [55,56]. An increase in TIMP1 gene expression and secretion has been shown in PDAC mouse models, as well as in human biopsies and serum [57]. The high serum concentration of TIMP1 is related to poor prognosis in ovarian cancer and in many other malignant tumors [57,58]. Our data also revealed a strong hyper-secretion of insulin-like growth factor-binding protein 1 (IGFBP1) in both R273H- and R175H-driven secretome samples. IGF signaling and p53 are strongly connected in cancer, especially in relation to tumor development and progression [59]. IGFBP1 is also involved in the activation of O-GlcNAcylation of FoxO1 in pancreatic β cells by promoting AKT inhibition [60] in cancer cell response to DNA damage [61] and it promotes angiogenesis in glioblastoma [62]. Furthermore, high levels of serum IGFBP-1 were shown in patients with nasopharyngeal carcinoma (NPC) and associated with poor prognosis [63]. In the secretome of both p53 mutants, we have also observed the hyper-secretion of the endothelial protein C receptor (PROCR), which is implicated in the carcinogenesis of various tumor types [64]. Indeed, PROCR has a tumor promoting effect and its silencing in gastric cancer inhibits the proliferation and migration via the ERK1/2 pathway, while, in ovarian cancer cells, it induces cell migration via MEK-ERK and Rho-GTPase pathways [65]. Interestingly, Wang et al. reported that IGF-1 receptor mediates the signaling function of PROCR in breast cancer cells [66]. In particular, they demonstrated that PROCR induces the activation of ERK and PI3K–AKT–mTOR signal through the transactivation of IGF-1R by Src and concomitantly stimulates RhoA–ROCK–p38 signals. In our system, we can hypothesize that the mutp53-dependent secretion of both IGFBP1 and PROCR might act in synergy to trigger intracellular pathways that are involved in cancer cell proliferation. Previous studies have suggested that PROCR^+^ cells have increased EMT features [67]. In our study, we observed that mutp53-dependent secretome increased the N/E cadherin ratio, which suggests the stimulation of EMT. Thus, PROCR might be another channel through which mutp53-dependent secretome promotes EMT and tumor progression. By a clinical perspective, PROCR can be considered to be a possible biomarker of cancer onset, since its secretion is related to enhanced cell survival, invasion, and immune down regulation in patients with ovarian cancer [68]. Dermcidin (DCD), ezrin (EZR), and prosaposin (PSAP) are also hyper-secreted in both R273H- and R175H-driven secretome samples and they promote cancer progression [69,70,71]. Elevated levels of DCD are associated with the early progression of breast cancer and metastatic progression of melanoma [72,73]. EZR promotes the invasion and metastasis of pancreatic cancer cells. It might provide a predictive and diagnostic signature in PDAC, but the mechanisms of ezrin-mediated tumor development still require further elucidation [70,74]. PSAP is a conserved glycoprotein with multiple functions and it is involved in the development of cancers [71,75]. In particular, in breast cancer, mesenchymal stem cells induces prosaposin secretion to drive the proliferation [76]. Furthermore, the serum-PSAP levels are increased in patients with advanced prostate cancer and this could provide a cell survival response after therapeutic interventions [77].

Our study also revealed hypo-secretion of five types of histones in both mutant R273H- and R175H-driven secretome samples. Besides nuclear functions, histones can act as endogenous danger signals when they shift from the nucleus to the extranuclear space [78]. Indeed, in response to apoptotic signals, histones translocate from genomic DNA to cytoplasm and they are then released in the extracellular compartment [79]. Thus, our data suggest that the reduced secretion of histones in mutp53-driven secretome samples might suggest one of several ways by which mutp53-driven secretome inhibits cell death-related phenomena, such as apoptosis, in accordance with the antiapoptotic role of intracellular mutant p53 isoforms [31].

## 5. Conclusions

Finally, these data show the key role of the *TP53* gene in the network of the differentially secreted proteins. We finally provide evidence that, in addition to the alteration of gene expression profile or to the specific protein-protein intracellular interactions, the oncogenic role of mutant p53 can also be due to a marked alteration of cancer secretome that can promote cancer aggressiveness in an autocrine/paracrine manner and regulate the cancer–stroma relationship. Future investigations are needed to further discover the role and biological impact in cancer microenvironment of the specific proteins that are differentially secreted by mutant p53 and identified in the present study. Furthermore, we will also aim to investigate whether some of these differentially secreted proteins may constitute a secreted signature detectable in PDAC patients’ sera that can be easily predictive for GOF *TP53* gene mutations in cancer patients. This might also enable the identification of targeted therapies that are specifically addressed to inhibit growth of PDACs carrying oncogenic mutant p53, which are strongly resistant to traditional chemotherapies.

## Figures and Tables

**Table 1:** Fifteen common secreted proteins by both R273H or R175H mutant p53 isoforms expressed in AsPC-1 cells and identified by using high-resolution SWATH-MS technology (*p* < 0.05).

Accession Name	Entry	Protein Names	Gene	Fold Change *versus* Mock (FC) R273H R175H
IBP1_HUMAN	P08833	Insulin-like growth factor-binding protein 1	IGFBP1	4.49 3.03
EPCR_HUMAN	Q9UNN8	Endothelial protein C receptor	PROCR	2.42 1.49
TIMP1_HUMAN	P01033	Metalloproteinase inhibitor 1	TIMP1	2.18 1.30
DCD_HUMAN	P81605	Dermcidin	DCD	1.94 1.37
EZRI_HUMAN	P15311	Ezrin	EZR	1.82 2.48
SAP_HUMAN	P07602	Prosaposin	PSAP	1.64 1.42
VIME_HUMAN	P08670	Vimentin	VIM	1.47 2.32
CSTN1_HUMAN	O94985	Calsyntenin-1	CLSTN1	1.43 2.51
TAD2B_HUMAN	Q86TJ2	Transcriptional adapter 2-beta	TADA2B	1.39 1.37
COF1_HUMAN	P23528	Cofilin-1	CFL1	1.35 1.52
H4_HUMAN	P62805	Histone H4	HIST1H4A	0.54 0.76
H31_HUMAN	P68431	Histone H3.1	HIST1H3A	0.43 0.46
H32_HUMAN	Q71DI3	Histone H3.2	HIST2H3A	0.41 0.40
H2AV_HUMAN	Q71UI9	Histone H2A.V	H2AFV	0.33 0.45
H2A2A_HUMAN	Q6FI13	Histone H2A type 2-A	HIST2H2AA3	0.32 0.45

## Supplementary Materials

The following are available online at https://www.mdpi.com/2218-273X/10/6/884/s1, Figure S1: p53 expression and functionality; Figure S2: Cell growth control both with and without fetal bovine serum; Figure S3: Secreted vimentin expression; Table S1: Secreted proteins by R175H-mutp53; Table S2: Secreted proteins by R273H-mutp53; Table S3: Analysis of secreted proteins by SignalP, SecretomeP software and in cancer secretome database; Table S4: Bioinformatic analyses of secreted proteins by DAVID software.
