# Supplementary material for: The Mutant p53-Driven Secretome Has Oncogenic Functions in Pancreatic Ductal Adenocarcinoma Cells
Source: Biomolecules. 2020 Jun 9;10(6):884. doi: 10.3390/biom10060884 (PMC7356389; doi:10.3390/biom10060884)
Supplement: Supplementary file 1 [file biomolecules-10-00884-s001.zip › biomolecules-807431 supplementary/supplementary.docx]

The mutant p53-Driven Secretome has Oncogenic Functions in Pancreatic Ductal Adenocarcinoma Cells

Giovanna Butera ^1^, Jessica Brandi ^2^, Chiara Cavallini ^3^, Aldo Scarpa ^4,5^, Rita T. Lawlor ^5^,
Maria Teresa Scupoli ^1,3^, Emilio Marengo ^6,7^, Daniela Cecconi ^2^, Marcello Manfredi ^7,8,^* and Massimo Donadelli ^1,^*

^1^ Department of Neurosciences, Biomedicine and Movement Sciences, Section of Biochemistry, University of Verona, Strada Le Grazie 8, 37134 Verona, Italy; [giovanna.butera@univr.it](mailto:giovanna.butera@univr.it) (G.B.); [mariateresa.scupoli@univr.it](mailto:mariateresa.scupoli@univr.it) (M.T.S.)

^2^ Department of Biotechnology, University of Verona, 37134 Verona, Italy; [jessica.brandi@univr.it](mailto:jessica.brandi@univr.it) (J.B.); [daniela.cecconi@univr.it](mailto:daniela.cecconi@univr.it) (D.C.)

^3^ Research Center LURM (Interdepartmental Laboratory of Medical Research), University of Verona, 37134 Verona, Italy; [chiara.cavallini@univr.it](mailto:chiara.cavallini@univr.it)

^4^ Department of Diagnostics and Public health, Section of Pathology, University of Verona, 37134 Verona, Italy; [aldo.scarpa@univr.it](mailto:aldo.scarpa@univr.it)

^5^ ARC-Net Centre for Applied Research on Cancer, University and Hospital Trust of Verona, 37134 Verona, Italy; [rita.lawlor@arc-net.it](mailto:rita.lawlor@arc-net.it)

^6^ Department of Sciences and Technological Innovation, University of Piemonte Orientale, 28100 Novara, Italy; [emilio.marengo@uniupo.it](mailto:emilio.marengo@uniupo.it)

^7^ Center for Translational Research on Autoimmune and Allergic Diseases, University of Piemonte Orientale, Italy; ISALIT, Spin-off at the University of Piemonte Orientale, 28100 Novara, Italy

^8^ Department of Translational Medicine, University of Piemonte Orientale, Italy, CAAD, corso Trieste 15/A, 28100 Novara, Italy

* Correspondence: marcello.manfredi@uniupo.it (M.M.); [massimo.donadelli@univr.it](mailto:massimo.donadelli@univr.it) (M.D.); Tel.: +39 0321660810 (M.M.); +39 045 8027281 (M.D.); Fax: +39 045 8027170 (M.D.)


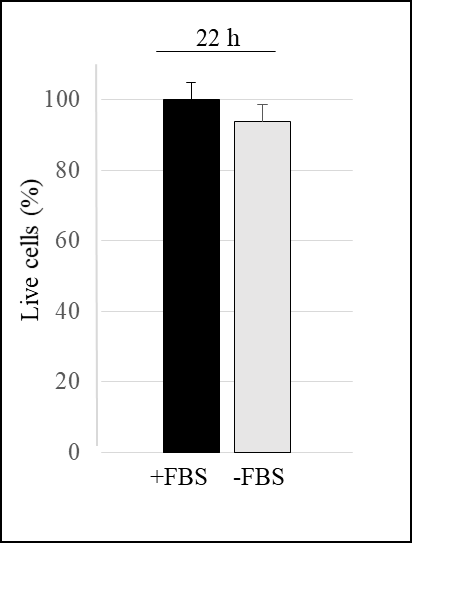


**Figure S1.** p53 expression and functionality. Expression of p53 was verified by Western blotting and the growth of H1299 cells stably expressing R273H mutant p53 was measured by Crystal Violet assay. ** *p* < 0.01 R273H vs Mock.


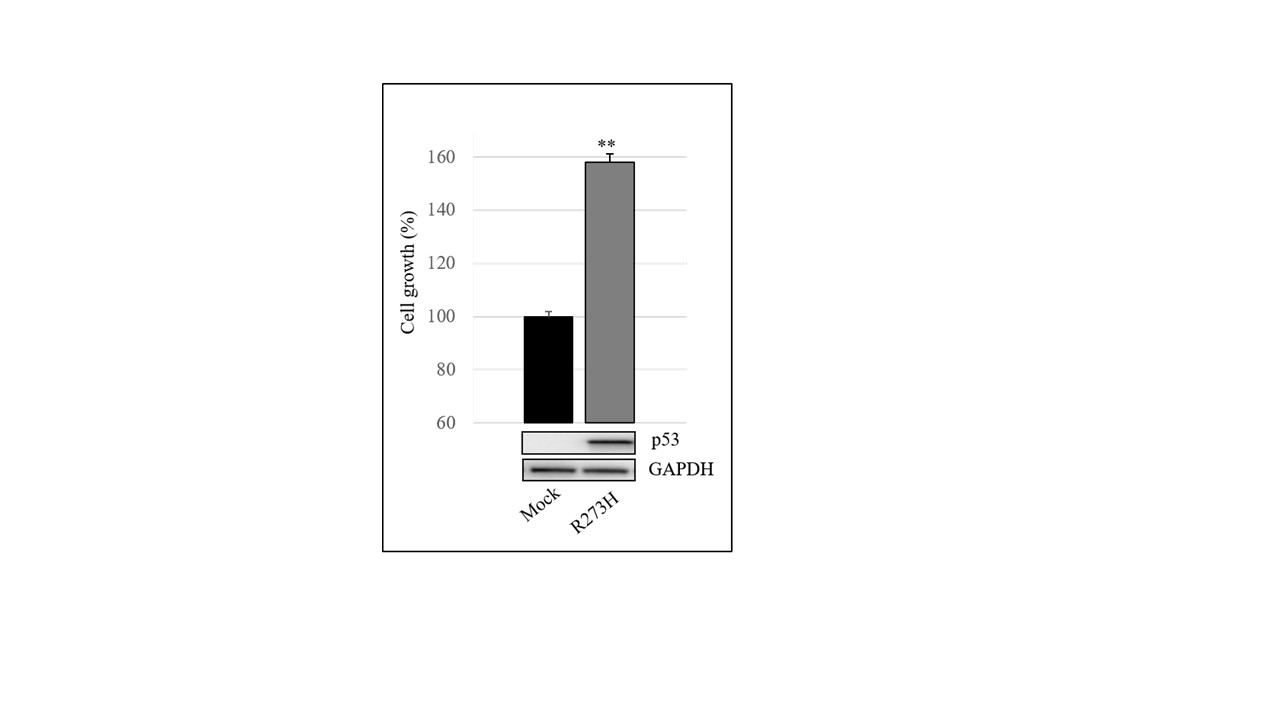


**Figure S2.** Cell growth control both with and without fetal bovine serum. AsPC-1 cells were grown in culture medium with or without fetal bovine serum (FBS) for 22 hours. Trypan blue exclusion assay was used to calculate the percentage of live cells. Cell counting was performed by automated cell counter.


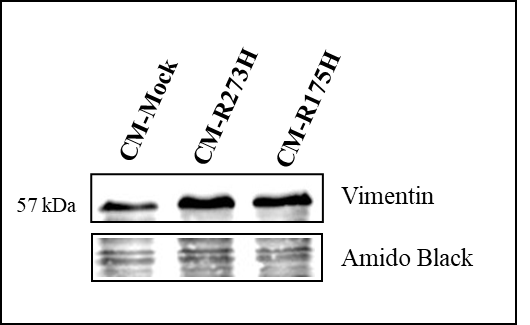


**Figure S3.** Secreted vimentin expression. Expression of vimentin was verified by Western blotting loading 70 μg of protein extracts derived from conditioned medium (CM) released by AsPC-1 cells expressing R273H and R175H mut p53 and probed with the indicated antibody. Amido Black staining was used as control loading.

**Table S1.** Secreted proteins by R175H-mutp53. Differentially secreted proteins in R175H-mutp53 AsPC-1 cells compared to mock (*p* < 0.05).

| **Accession name** | **Entry** | **Protein names** | **Gene** | **FC** |
| --- | --- | --- | --- | --- |
| IBP1_HUMAN | P08833 | Insulin-like growth factor-binding protein 1 | IGFBP1 | 3.03 |
| CSTN1_HUMAN | O94985 | Calsyntenin-1 | CLSTN1 | 2.51 |
| EZRI_HUMAN | P15311 | Ezrin | EZR | 2.48 |
| VIME_HUMAN | P08670 | Vimentin | VIM | 2.32 |
| CH60_HUMAN | P10809 | 60 kDa heat shock protein, mitochondrial | HSPD1 | 1.72 |
| NPM_HUMAN | P06748 | Nucleophosmin | NPM1 | 1.65 |
| PRDX1_HUMAN | Q06830 | Peroxiredoxin-1 | PRDX1 | 1.61 |
| KPYM_HUMAN | P14618 | Pyruvate kinase PKM | PKM | 1.56 |
| 1433Z_HUMAN | P63104 | 14-3-3 protein zeta/delta | YWHAZ | 1.56 |
| LDHA_HUMAN | P00338 | L-lactate dehydrogenase A chain | LDHA | 1.54 |
| MDHM_HUMAN | P40926 | Malate dehydrogenase, mitochondrial | MDH2 | 1.54 |
| COF1_HUMAN | P23528 | Cofilin-1 | CFL1 | 1.52 |
| G3P_HUMAN | P04406 | Glyceraldehyde-3-phosphate dehydrogenase | GAPDH | 1.52 |
| S10A4_HUMAN | P26447 | Protein S100-A4 | S100A4 | 1.52 |
| MYH9_HUMAN | P35579 | Myosin-9 | MYH9 | 1.50 |
| ROA2_HUMAN | P22626 | Heterogeneous nuclear  ribonucleoproteins A2/B1 | HNRNPA2B1 | 1.49 |
| EPCR_HUMAN | Q9UNN8 | Endothelial protein C receptor | PROCR | 1.49 |
| S10A6_HUMAN | P06703 | Protein S100-A6 | S100A6 | 1.43 |
| HS90A_HUMAN | P07900 | Heat shock protein HSP 90-alpha | HSP90AA1 | 1.43 |
| ACTB_HUMAN | P60709 | Actin, cytoplasmic 1 | ACTB | 1.42 |
| SAP_HUMAN | P07602 | Prosaposin | PSAP | 1.42 |
| 1433G_HUMAN | P61981 | 14-3-3 protein gamma | YWHAG | 1.42 |
| HSPB1_HUMAN | P04792 | Heat shock protein beta-1 | HSPB1 | 1.40 |
| HSP7C_HUMAN | P11142 | Heat shock cognate 71 kDa protein | HSPA8 | 1.39 |
| THIO_HUMAN | P10599 | Thioredoxin | TXN | 1.37 |
| DCD_HUMAN | P81605 | Dermcidin | DCD | 1.37 |
| TAD2B_HUMAN | Q86TJ2 | Transcriptional adapter 2-beta | TADA2B | 1.37 |
| SETLP_HUMAN | P0DME0 | Protein SETSIP | SETSIP | 1.35 |
| S10AB_HUMAN | P31949 | Protein S100-A11 | S100A11 | 1.34 |
| PROF1_HUMAN | P07737 | Profilin-1 | PFN1 | 1.33 |
| TPIS_HUMAN | P60174 | Triosephosphate isomerase | TPI1 | 1.31 |
| TIMP1_HUMAN | P01033 | Metalloproteinase inhibitor 1 | TIMP1 | 1.30 |
| IBP6_HUMAN | P24592 | Insulin-like growth factor-binding protein 6 | IGFBP6 | 0.77 |
| H4_HUMAN | P62805 | Histone H4 | HIST1H4A | 0.76 |
| CP2R1_HUMAN | Q6VVX0 | Vitamin D 25-hydroxylase | CYP2R1 | 0.73 |
| TRFL_HUMAN | P02788 | Lactotransferrin | LTF | 0.73 |
| MK_HUMAN | P21741 | Midkine | MDK | 0.73 |
| CAPG_HUMAN | P40121 | Macrophage-capping protein | CAPG | 0.65 |
| SDC4_HUMAN | P31431 | Syndecan-4 | SDC4 | 0.62 |
| NGAL_HUMAN | P80188 | Neutrophil gelatinase-associated lipocalin | LCN2 | 0.60 |
| H31_HUMAN | P68431 | Histone H3.1 | HIST1H3A | 0.46 |
| H2AV_HUMAN | Q71UI9 | Histone H2A.V | H2AFV | 0.45 |
| H2A2A_HUMAN | Q6FI13 | Histone H2A type 2-A | HIST2H2AA3 | 0.45 |
| H32_HUMAN | Q71DI3 | Histone H3.2 | HIST2H3A | 0.40 |
| NCOA6_HUMAN | Q14686 | Nuclear receptor coactivator 6 | NCOA6 | 0.31 |

**Table S2.** Secreted proteins by R273H-mutp53. Differentially secreted proteins in R273H-mutp53 AsPC-1 cells compared to mock (*p* < 0.05).

| **Accession name** | **Entry** | **Protein names** | **Gene** | **FC** |
| --- | --- | --- | --- | --- |
| EFTU_HUMAN | P49411 | Elongation factor Tu, mitochondrial | TUFM | 5.78 |
| IBP1_HUMAN | P08833 | Insulin-like growth factor-binding protein 1 | IGFBP1 | 4.49 |
| TPM1_HUMAN | P09493 | Tropomyosin alpha-1 chain | TPM1 | 2.58 |
| EPCR_HUMAN | Q9UNN8 | Endothelial protein C receptor | PROCR | 2.42 |
| MMP7_HUMAN | P09237 | Matrilysin | MMP7 | 2.36 |
| TIMP2_HUMAN | P16035 | Metalloproteinase inhibitor 2 | TIMP2 | 2.32 |
| TIMP1_HUMAN | P01033 | Metalloproteinase inhibitor 1 | TIMP1 | 2.18 |
| IBP6_HUMAN | P24592 | Insulin-like growth factor-binding protein 6 | IGFBP6 | 2.11 |
| RNT2_HUMAN | O00584 | Ribonuclease T2 | RNASET2 | 2.09 |
| PPGB_HUMAN | P10619 | Lysosomal protective protein | CTSA | 1.97 |
| LG3BP_HUMAN | Q08380 | Galectin-3-binding protein | LGALS3BP | 1.95 |
| RAB15_HUMAN | P59190 | Ras-related protein Rab-15 | RAB15 | 1.94 |
| DCD_HUMAN | P81605 | Dermcidin | DCD | 1.94 |
| EZRI_HUMAN | P15311 | Ezrin | EZR | 1.82 |
| GNS_HUMAN | P15586 | N-acetylglucosamine-6-sulfatase | GNS | 1.80 |
| FAM3C_HUMAN | Q92520 | Protein FAM3C | FAM3C | 1.78 |
| PGBM_HUMAN | P98160 | Basement membrane-specific heparan sulfate proteoglycan core protein | HSPG2 | 1.76 |
| CAD17_HUMAN | Q12864 | Cadherin-17 | CDH17 | 1.76 |
| HMGA1_HUMAN | P17096 | High mobility group protein HMG-I/HMG-Y | HMGA1 | 1.70 |
| CSPG2_HUMAN | P13611 | Versican core protein | VCAN | 1.70 |
| CATB_HUMAN | P07858 | Cathepsin B | CTSB | 1.66 |
| SAP_HUMAN | P07602 | Prosaposin | PSAP | 1.64 |
| BGH3_HUMAN | Q15582 | Transforming growth factor-beta-induced protein ig-h3 | TGFBI | 1.64 |
| CP2R1_HUMAN | Q6VVX0 | Vitamin D 25-hydroxylase | CYP2R1 | 1.63 |
| GDN_HUMAN | P07093 | Glia-derived nexin | SERPINE2 | 1.62 |
| APLP2_HUMAN | Q06481 | Amyloid-like protein 2 | APLP2 | 1.61 |
| AGRIN_HUMAN | O00468 | Agrin | AGRN | 1.60 |
| NBL1_HUMAN | P41271 | Neuroblastoma suppressor of  tumorigenicity 1 | NBL1 | 1.58 |
| TRFE_HUMAN | P02787 | Serotransferrin | TF | 1.51 |
| B2MG_HUMAN | P61769 | Beta-2-microglobulin | B2M | 1.50 |
| ALBU_HUMAN | P02768 | Serum albumin | ALB | 1.48 |
| VIME_HUMAN | P08670 | Vimentin | VIM | 1.47 |
| CSTN1_HUMAN | O94985 | Calsyntenin-1 | CLSTN1 | 1.43 |
| TAD2B_HUMAN | Q86TJ2 | Transcriptional adapter 2-beta | TADA2B | 1.39 |
| TRFL_HUMAN | P02788 | Lactotransferrin | LTF | 1.37 |
| SDC1_HUMAN | P18827 | Syndecan-1 | SDC1 | 1.37 |
| COF1_HUMAN | P23528 | Cofilin-1 | CFL1 | 1.35 |
| NCOA6_HUMAN | Q14686 | Nuclear receptor coactivator 6 | NCOA6 | 1.32 |
| HS90B_HUMAN | P08238 | Heat shock protein HSP 90-beta | HSP90AB1 | 0.69 |
| NUCL_HUMAN | P19338 | Nucleolin | NCL | 0.69 |
| 1433Z_HUMAN | P63104 | 14-3-3 protein zeta/delta | YWHAZ | 0.68 |
| IF4A1_HUMAN | P60842 | Eukaryotic initiation factor 4A-I | EIF4A1 | 0.67 |
| MDHM_HUMAN | P40926 | Malate dehydrogenase, mitochondrial | MDH2 | 0.66 |
| FLNB_HUMAN | O75369 | Filamin-B | FLNB | 0.56 |
| H12_HUMAN | P16403 | Histone H1.2 | HIST1H1C | 0.56 |
| H4_HUMAN | P62805 | Histone H4 | HIST1H4A | 0.54 |
| EF2_HUMAN | P13639 | Elongation factor 2 (EF-2) | EEF2 | 0.53 |
| ACTB_HUMAN | P60709 | Actin, cytoplasmic 1 | ACTB | 0.51 |
| CH60_HUMAN | P10809 | 60 kDa heat shock protein, mitochondrial | HSPD1 | 0.49 |
| CALR_HUMAN | P27797 | Calreticulin | CALR | 0.48 |
| MYH9_HUMAN | P35579 | Myosin-9 | MYH9 | 0.44 |
| MPCP_HUMAN | Q00325 | Phosphate carrier protein, mitochondrial | SLC25A3 | 0.44 |
| DESP_HUMAN | P15924 | Desmoplakin | DSP | 0.43 |
| H31_HUMAN | P68431 | Histone H3.1 | HIST1H3A | 0.43 |
| H32_HUMAN | Q71DI3 | Histone H3.2 | HIST2H3A | 0.41 |
| H2AJ_HUMAN | Q9BTM1 | Histone H2A.J | H2AFJ | 0.40 |
| H2AV_HUMAN | Q71UI9 | Histone H2A.V | H2AFV | 0.33 |
| H2A2A_HUMAN | Q6FI13 | Histone H2A type 2-A | HIST2H2AA3 | 0.32 |
